# 

**Published:** 1897-12-11

**Authors:** 


					\
the HOSPITAL. ABSOLUTELY PURE, therefore BEST. Dec. 11th, 1897.
CADBURY'S ?a ww CADBURY'S
OOCOA
''Sw?Si.d?!f.!S:!'P?r','7"P,eSe" B> mm HO ALKALIES USED.
OOOOA
?-.I'.
<Clascon Western Inh'rmary
No. 585,'Vol. XXHE. CfaSSSJ^] Saturday,
Dec. 11th, 1897.
'NORFOLKaho NORWICH HOSPITAL
L Bee" p. 182. J
fSubsoription Terms,-] TWOPENCE.

				

